# The Role of Platelets in Pulmonary Hypertension: From Activation to Pulmonary Vascular Remodeling—A Review Article

**DOI:** 10.3390/biomedicines14061203

**Published:** 2026-05-27

**Authors:** Patrycja Wszelaki, Aleksandra Karczmarska, Szymon Szymoniuk, Grzegorz Grześk, Zbigniew Włodarczyk, Joanna Sikora

**Affiliations:** 1 Research and Education Unit for Experimental Biotechnology, Department of Transplantology and General Surgery, Collegium Medicum, Nicolaus Copernicus University, 85-094 Bydgoszcz, Poland; wszelakipatrycja@cm.umk.pl (P.W.);; 2 Department of Cardiology and Clinical Pharmacology, Collegium Medicum, Nicolaus Copernicus University, 85-168 Bydgoszcz, Poland; g.grzesk@cm.umk.pl; 3 Department of Transplantology and General Surgery, Collegium Medicum, Nicolaus Copernicus University, 85-094 Bydgoszcz, Poland

**Keywords:** pulmonary arterial hypertension, platelets, vascular inflammation, metabolic reprogramming, platelet–leukocyte aggregates, platelet paradox

## Abstract

The article presents the results of a structured literature review of the past decade and focuses on the crucial role of platelets in the pathogenesis of pulmonary arterial hypertension (PAH). It explores how endothelial dysfunction initiates early prothrombotic signals that activate platelets, which, in response, adopt a pro-inflammatory phenotype, release cytokines and chemokines, and form aggregates with leukocytes, thereby modulating their migration and activity. A key feature of PAH is the “platelet paradox,” in which chronic in vivo activation coexists with reduced ex vivo reactivity due to functional exhaustion. Prolonged stimulation and disease progression lead to complex hemostatic dysregulation, characterized by heterogeneity in platelet phenotypes. At the same time, platelets undergo immunometabolic reprogramming, with a predominance of glycolysis, over oxidative phosphorylation, mitochondrial dysfunction, altered fatty acid oxidation (FAO), increased lactate production, and enhanced vesicle release. These phenomena sustain inflammation and promote pulmonary vascular remodeling. This study aims to review the current mechanisms of immunometabolic platelet activation in pulmonary arterial hypertension. It primarily focuses on platelet aspects as key elements in disease progression and as potential sources of new biomarkers and therapeutic targets.

## 1. Introduction

Despite current therapies, pulmonary arterial hypertension (PAH) remains a progressive and fatal condition, highlighting an urgent need for treatments targeting vascular remodeling. While platelets are traditionally viewed through the lens of thrombosis, recent studies demonstrate their active role in immune and metabolic pathways. While platelets have traditionally been viewed in the context of thrombosis, emerging evidence indicates that they function as critical regulators of immunometabolic signaling. However, their role as immunometabolic mediators driving progressive vascular fibrosis and remodeling remains incompletely defined. Moreover, there is a distinct gap in understanding how specific metabolic reprogramming of platelets correlates with PAH severity and whether these alterations could serve as early markers of irreversible pulmonary vascular remodeling.

While the role of PAH has been previously established by Cullivan, Murphy, and Grześk, this article offers a targeted synthesis of emerging evidence regarding the immunometabolic reprogramming of platelets. We aim to bridge the gap between aberrant platelet bioenergetics, specifically shifts in glycolysis and fatty acid oxidation, and their direct contribution to pulmonary vascular remodeling. In doing so, we provide a novel perspective that transcends traditional hemostatic and pharmacological frameworks. This review analyzes the role of platelets in PAH pathogenesis, with a particular focus on the mechanisms driving their immunometabolic activation. We systematically trace the progression from endothelial injury and prothrombotic signaling to the acquisition of a pro-inflammatory phenotype and platelet–immune cell interactions, ultimately elucidating their impact on structural remodeling. Furthermore, we identify potential biomarkers and novel therapeutic avenues arising from platelet dysfunction.

Subsequent sections address methodology; endothelial injury and early prothrombotic signals; inflammation-induced platelet activation; the immunothrombotic role of platelets; hemostatic dysfunction of hyperactive platelets; platelet immunometabolic reprogramming; vascular remodeling; clinical relevance; and future directions.

## 2. Methodology

**Figure 1:**
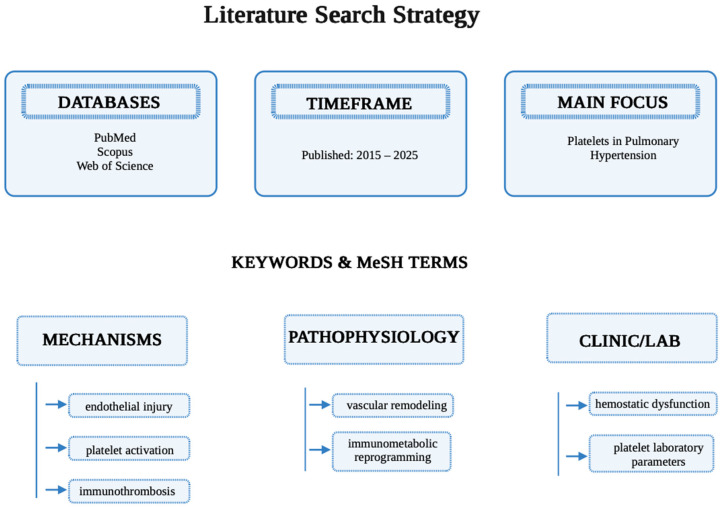
Literature search strategy.

A structured literature search was conducted in PubMed, Scopus, and Web of Science for articles published between 2015 and 2025. The search strategy combined keywords and MeSH terms related to platelets in pulmonary hypertension, including the following: endothelial injury, platelet activation and immunothrombotic role, platelet hemostatic dysfunction, platelet immunometabolic reprogramming, vascular remodeling, and laboratory parameters of platelets, including hemostasis tests. Reference lists of relevant articles were also investigated to identify additional studies not captured in the primary database search (Figure 1).

The following inclusion and exclusion criteria were applied. Inclusion criteria included (1) original research articles, clinical studies, and reviews addressing the role of platelets in the pathogenesis of PAH, with particular focus on molecular, inflammatory, and metabolic mechanisms; (2) studies published within the last 10 years (2015–2025) to ensure that the data were current; and (3) publications in English with full-text availability. Exclusion criteria included (1) case reports lacking analytical contribution to the understanding of general pulmonary hypertension pathophysiology, as well as studies with small sample sizes insufficient to support reliable statistical conclusions and (2) articles without full-text access or published in languages other than English.

After removing the duplicates, the titles and abstracts were independently screened by two reviewers (PW, JS) to assess relevance. Full texts of potentially relevant articles were retrieved and critically evaluated for methodological quality, relevance to PAH, and the representativeness of the presented molecular mechanism relative to contemporary concepts of immunometabolism. Given the narrative nature of the review, a formal systematic quality assessment or meta-analysis was not performed; instead, studies were included based on their ability to provide mechanistic insights, translational relevance, or clinical context.

During the preparation of the manuscript, the authors used Google Gemini (version Gemini Flash 3) to ensure that the references were formatted according to the journal guidelines, create the graphical abstract, and assist with the literature search. Additionally, BioRender.com was used to generate the graphics included in the manuscript, and Grammarly was used for language editing and proofreading. The authors have reviewed and edited the manuscript and take full responsibility for the content of this publication.

## 3. Endothelial Injury and Early Prothrombotic Signals

One of the factors contributing to pathological vascular remodeling is an imbalance between pro-inflammatory mediators and vasoactive factors released by pulmonary artery endothelial cells (PAECs). The inflammatory process leads to endothelial cell (EC) dysfunction, disrupting their normal synthesis and secretion of mediators and cytokines. This enhances the local inflammatory response and promotes excessive proliferation and migration of ECs and adjacent vascular cells. Patients with PAH exhibit elevated levels of pro-inflammatory cytokines, such as interleukin-1 (IL-1) and interleukin-6 (IL-6), chemokines, and adhesion molecules in lung tissue and in activated ECs [1]. According to Yijie Hu et al., lung biopsies from patients with PAH revealed virtually all types of inflammatory cells in the vicinity of the remodeled vascular system, mainly including macrophages, mast cells, T and B lymphocytes, dendritic cells, and neutrophils [2]. Furthermore, studies have shown that neutrophils play a key role in the early stages of PAH by releasing the protein S100A9, which activates the RAGE/PI3K/AKT pathway in ECs, thereby inducing endothelial dysfunction and contributing to pulmonary vascular remodeling. Some authors suggest that reducing neutrophil numbers may inhibit disease progression and thereby represent a potential therapeutic target [3]. As a result, EC dysfunction and activation not only exacerbate inflammation but also promote early prothrombotic vascular activation, contributing to the initiation of pulmonary vascular remodeling and the progression of PAH [1].

In conditions of progressive inflammation in PAH, hypoxia becomes an important factor that intensifies endothelial damage, leading to excessive production of reactive oxygen species (ROS). Superoxide anion (O_2_^−^) and hydrogen peroxide (H_2_O_2_) act as important signaling mediators. They activate pathways that regulate pulmonary vascular tension, cell proliferation and apoptosis, inflammatory response, and fibrosis. However, when ROS concentrations (H_2_O_2_ especially) exceed physiological norms, endothelial dysfunction intensifies, its anticoagulant properties are lost, and pathophysiological vasoconstrictive reactions are induced, promoting the development and progression of PAH [4].

Alveolar hypoxia, a key feature of high-altitude exposure, induces acute pulmonary vasoconstriction and contributes to vascular remodeling, persistent pulmonary hypertension, and right ventricular overload, which may ultimately progress to right ventricular failure. It is known that oxygen detection and signal transmission occur primarily in the smooth muscle cells (SMCs) of the precapillary arteries. Under hypoxic conditions, excessive mitochondrial ROS production increases intracellular Ca^2+^ concentration in pulmonary artery smooth muscle cells (PASMCs), promoting vasoconstriction and early prothrombotic hemostatic changes [5].

Pulmonary hypertension is driven by impaired endothelial regulation of vascular tone, resulting in a disturbed balance between vasoconstriction and vasodilation. Under physiological conditions, endothelial cells maintain low pulmonary arterial pressure through coordinated release of vasoactive mediators. Endothelial dysfunction in this area leads to changes in the vascular environment, promoting prothrombotic signals and shifting the vascular balance toward a state that favors intravascular thrombosis [6,7].

In PAH, impaired vasodilation is primarily due to reduced production of key vasodilators, such as nitric oxide (NO) and prostacyclin. NO, a fast-acting molecule, is responsible for vasodilation, has anticoagulant properties, and regulates EC proliferation. According to Nowaczyk et al., stimulation of the NO pathway, due to its biochemical properties and documented clonal activity, constitutes an important therapeutic target in [8]. Prostacyclin, synthesized in the endothelium by prostacyclin synthase, also has anticoagulant properties and inhibits cell proliferation; reduced concentrations in PAH promote vasoconstriction, SMC proliferation, and an increased tendency to thrombosis [6]. PAH is associated with an abnormal regulation of the fundamental vasoconstrictor endothelin-1 (ET-1), further disrupting endothelial function and promoting disease progression. The highest concentration of ET-1 is found in lung tissue. Its overexpression, together with increased receptor numbers, amplifies prothrombotic signals and perpetuates vasoconstriction. PAH is also characterized by upregulated expression of vascular endothelial growth factor (VEGF) and its receptor, VEGF receptor 2 (VEGFR2). These factors have a protective effect in the early stages of PAH but subsequently promote abnormal endothelial proliferation [6].

Disorders include reduced prostacyclin levels, increased ET-1 expression, impaired NO synthesis and signaling within the NO–sGC–cGMP pathway, promoting pulmonary artery constriction. To date, three main therapeutic pathways have been studied in detail: prostacyclin, NO, and ET-1 [7,9]. Endothelin receptor antagonists limit the action of ET-1, and phosphodiesterase type 5 (PDE-5) inhibitors inhibit cyclic guanosine monophosphate (cGMP) degradation, while soluble guanylate cyclase (sGC) stimulators increase its production, resulting in vasodilation and the inhibition of SMC proliferation in the lungs [9].

The PATENT-1 and CHEST-1 projects advanced the understanding of the NO pathway, demonstrating that sGC stimulation and the subsequent increase in cGMP induces vasodilation and inhibits pathological vascular remodeling, which is essential for maintaining vascular homeostasis. This has led to the introduction of modern sGC stimulators into clinical practice, such as riociguat for the treatment of chronic thromboembolic pulmonary hypertension (CTEPH) and vericiguat, approved in 2021, for heart failure management [10]. Fellows et al. developed miniature three-dimensional engineered pulmonary artery tissues (EPATs) to model human pulmonary artery contractility and enable high-throughput screening of vasoactive compounds [11].

## 4. Inflammation-Induced Platelet Activation

Processes such as excessive vasoconstriction, vascular wall remodeling, and in situ thrombosis are major contributors in the pathophysiology of PAH and are closely related to platelet activity and the coagulation cascade. To fully comprehend platelet dysregulation in PAH, it is essential to define the gasotransmitter pathway, as a fundamental molecular background [12]. The NO/CO-sGC-cGMP pathway serves as a common regulator of PAECs, PASMCs, and platelets [13]. Under physiological conditions, endothelium-derived NO and carbon monoxide (CO) diffuse into platelets, activating sGC to produce cGMP. This intraplatelet signaling cascade potently inhibits platelet activation and aggregation. In PAH, severe PAEC dysfunction leads to a deficiency in these gasotransmitters, depriving platelets of their natural inhibitory signals and shifting the balance toward a prothrombotic phenotype [14].

Published data indicate that platelets in patients with PAH are chronically activated and exhibit functional abnormalities, while their procoagulant activity may be reduced due to prolonged stimulation of the coagulation system [15,16]. This observation introduces the concept of the “platelet paradox” [17], a phenomenon central to understanding PAH pathophysiology, in which platelets are chronically activated in vivo (as evidenced by elevated activation markers) yet demonstrate reduced aggregation capacity and diminished reactivity in ex vivo functional assays. This paradox arises from persistent prothrombotic stress leading to functional exhaustion and secondary depletion of granule contents. Consequently, standard platelet function tests may underestimate the true extent of in vivo platelet activation in PAH, with significant implications for diagnostic interpretation and therapeutic decision-making. According to Vrigkou et al., although the patient’s clinical condition favors thrombus formation, clinical tests often reveal a blunted platelet response. This is directly related to chronic platelet activation, as platelets are heavily involved in the pathogenic process of PAH; as a result, their reactivity becomes depleted [18]. The same authors demonstrated a significant increase in platelet activation markers, such as serotonin and thromboxane (TXA), in patients with PAH compared to the control group [16], confirming the serotonin hypothesis of PAH [19] and elevated TXA levels [20]. Increased concentrations of P-selectin, a marker of platelet activation and endothelial damage, have also been documented [16,19]. Endothelial damage and prothrombotic signals create an environment conducive to sustained platelet activation in the inflammatory course of PAH. The relationship between inflammation and platelet activation was confirmed by Delaney et al., who observed increased platelet numbers and hypoxia-induced inflammation, resulting in a twofold increase in CD41 expression in the lungs of patients with idiopathic PAH (IPAH) [21].

Platelet aggregation, a fundamental element of primary hemostasis, is impaired in patients with PAH, consistent with the platelet paradox described above. In addition to reduced maximum aggregation, platelet aggregates in this population are significantly less stable than those in healthy individuals [15]. Research by Virgkou et al. demonstrated that patients with primary diagnosed PAH exhibited reduced platelet aggregation, decreased clot propagation, and limited thrombin generation, as well as a delayed initiation of the coagulation process [15,16]. In cases of acute decompensation of pulmonary hypertension, platelet aggregation is observed in the lungs [22]. In CTEPH, an inflammatory-immune disorder, the formation of platelet and monocyte aggregates is gaining increasing importance [23]. Sun et al. demonstrated that platelet activation and aggregation in CTEPH promote the formation of neutrophil extracellular traps (NETs), which accelerate disease progression and prolong thrombus duration [24]. Activated platelets stimulate NET formation by neutrophils, and NETs in turn provide a scaffold for further platelet adhesion and thrombus propagation, creating a positive feedback loop that sustains immunothrombosis in the pulmonary vasculature [24]. Changes indicating abnormal platelet function in primary hemostasis can already be observed in routine laboratory parameters such as mean platelet volume (MPV) and platelet distribution width (PDW) [25].

Extended hemostasis diagnostics use specialized tests to assess platelet activation and function [26]. These include light transmission aggregometry (LTA) with epinephrine (EPI) and adenosine diphosphate (ADP) agonists and rotational thromboelastometry (ROTEM), which enable detailed analysis of primary hemostasis and the coagulation process [18]. Studies indicate that patients with CTEPH exhibit reduced platelet aggregation and stability, prolonged times to clot initiation and propagation, delayed fibrinolysis, and impaired thrombin generation. The reduced ability of platelets to aggregate may result from their chronic activation, leading to prolonged release of granule contents and secondary depletion of reactivity (a manifestation of the platelet paradox) [18,27]. In addition, patients exhibit reduced clot lysis rates, as confirmed by data indicating fibrin resistance to degradation in CTEPH and platelet clot instability, characterized by a prolonged time to reach target clot strength [18,28].

## 5. Immunothrombotic Role of Platelets

Growing evidence supports the central involvement of immunothrombotic mechanisms driven by dynamic interactions between platelets and immune cells in the pathophysiology of PAH. Activated platelets serve as key mediators of communication among hemostasis, inflammation, and the immune response, forming complex signaling and structural networks involving platelets, leukocytes, and components of the coagulation system [24].

Research by Åberg et al. has shown that patients with PAH and CTEPH experience increased platelet activation in the circulation, accompanied by increased formation of platelet–leukocyte aggregates involving both monocytes and neutrophils. These observations confirm that PAH is characterized by a systemic inflammatory component and that the formation of platelet–monocyte and platelet–neutrophil complexes may be a mechanism that promotes and sustains this chronic inflammation [29].

In inflammatory and thrombotic diseases, platelets show a marked ability to interact with circulating leukocytes, particularly monocytes and neutrophils. The formation of leukocyte–platelet aggregates is initiated by P-selectin expression on activated platelets, whose concentration is significantly elevated in patients with PAH. Importantly, monocyte–platelet aggregates demonstrate greater diagnostic sensitivity for platelet activation than P-selectin levels alone, highlighting their importance as an immunothrombotic biomarker in PAH [30].

Beyond their role in aggregate formation, activated platelets in PAH serve as a significant source of pro-inflammatory mediators. Upon activation, platelets release α-granule contents that recruit and activate leukocytes at sites of vascular injury [27]. Furthermore, large amounts of platelet-derived CD40L are released from activated platelets, thereby inducing pro-inflammatory mediators production, immune cell recruitment, and SMC proliferation and hypertrophy [14]. sCD40L engages CD40 on ECs and monocytes, amplifying the expression of adhesion molecules, tissue factor (TF), and additional cytokines [14,17]. This platelet-driven inflammatory milieu transforms the pulmonary vascular environment from a site of localized thrombosis into a sustained inflammatory niche that drives remodeling [17,27].

Another important aspect of the immunothrombotic pathology of PAH is the observation that platelets not only form microthrombi in the lumen of the vessels but also accumulate within the wall and adventitia of the remodeled pulmonary arteries. Platelets have been found to co-localize with leukocytes, especially monocytes, suggesting a potential role in local inflammatory processes and vascular remodeling in patients with PAH [31]. The increased presence of platelets in the wall space and in the lumen of distal muscular arteries suggests that they participate in mechanisms beyond classical thrombosis and mechanical flow disturbances in pulmonary circulation.

It remains unclear whether monocytes recruited to altered pulmonary arteries exhibit primary functional abnormalities that promote their migration. However, available data indicate that monocytes in IPAH exhibit a distinct activation profile, characterized by reduced expression of bone morphogenetic protein receptor type 2 (BMPR2) and CD14, with a concurrent increase in STAT1–IFN axis activity. This phenotype is associated with impaired regulation of monocyte maturation and activation, leading to increased invasiveness in the pulmonary circulation [32]. Consequently, altered monocyte bioactivity may promote increased recruitment of these cells to pulmonary vessels, amplifying local inflammatory processes and supporting the progression of vascular changes characteristic of PAH.

Recent studies suggest that advanced analytical methods facilitate a deeper understanding of the immunological aspects of PAH. Yang et al. demonstrated that data integration using machine learning enables the development of an immune cell signature useful for diagnosing pulmonary hypertension [33]. In addition, Wu et al. used single-cell analysis of peripheral blood in patients with high-altitude hypoxia-induced PAH to identify a distinct monocyte phenotype, suggesting modulation of leukocyte function in specific disease conditions [34]. The described monocyte reprogramming not only intensifies local inflammation but also serves as a critical nexus between the immune system and hemostatic control. Specifically, the phenotypic shift driven by the STAT1–IFN axis modulates platelet activation, predisposing them to a prothrombotic state. This tight interplay between immunothrombosis and hemostatic dysregulation explains how chronic immune overstimulation ultimately leads to functional platelet exhaustion, explored in detail in the following section.

## 6. Hemostatic Dysfunction of Hyperactive Platelets

The assessment of platelet function in PAH reveals a complex “platelet paradox,” as described in Section 4, characterized by simultaneous in vivo chronic activation and altered ex vivo reactivity [17]. Clinical evidence supports this dysregulation; patients with PAH exhibit significantly elevated markers such as serotonin and TXA, confirming the serotonin hypothesis and persistent prothrombotic signaling [16,17,18,19,20]. Furthermore, increased CD41 expression in the lungs of patients with IPAH suggests that hypoxia-induced inflammation is a key driver of this state [21]. Importantly, this phenomenon was further analyzed by Vrigkou et al., confirming that chronic procoagulant activation can lead to progressive impairment of platelet aggregation capacity, suggesting functional exhaustion and loss of normal reactivity under prolonged prothrombotic stress conditions [18].

Data on platelet-derived microparticles (PDMPs) indirectly confirm the hypothesis that chronic activation of the coagulation system leads to the subsequent depletion of procoagulant reserves. PDMPs, released during platelet activation, exhibit strong prothrombotic properties. Elevated levels are found in patients with PAH, particularly in various subtypes such as CTEPH or IPAH, compared to the control group [35]. Furthermore, PDMPs exhibit 5–100-fold greater procoagulant activity than normal platelet surfaces, thereby promoting thrombin formation by exposing phosphatidylserine. These findings are consistent with the persistent hypercoagulability observed in PAH, including increased TF expression, elevated von Willebrand factor (vWF) concentrations, and platelet aggregation disorders [36]. To summarize the issue of hemostatic dysfunction in hyperactive platelets, PDMPs are products of activated platelets; they can therefore be considered potential biomarkers of their pathological activation [37].

Finally, the interpretation of these profiles further complicates the identification of PAH-specific pharmacotherapy. Prostacyclin analogs, a cornerstone of treatment, are potent inhibitors of platelet activation via the cAMP pathway. Accordingly, the hyporeactivity observed in functional assays may reflect not only pathophysiological exhaustion but also direct pharmacological modulation of platelet signaling.

## 7. Platelet Immunometabolic Reprogramming

In recent years, scientific reports on the etiopathogenesis of PAH have highlighted metabolic dysregulation and cellular reprogramming. Changes in cell metabolism and bioenergetics are more commonly described in the literature, with mitochondria identified as a critical determinant of this process, constituting a universal feature of PAH. Importantly, these metabolic alterations are not confined to resident vascular cells; circulating platelets also undergo profound immunometabolic reprogramming, directly driving their pro-inflammatory and pro-remodeling activities [38,39]. In addition to highlighting alternative energy sources in PAH, this reprogramming involves dysfunctional mitochondrial processes [40,41]. The literature indicates that discoveries in cellular reprogramming may yield new therapeutic targets by modulating metabolism [42,43].

### 7.1. Glycolytic Reprogramming

Macrophages and dendritic cells, upon activation by pro-inflammatory signals, undergo a swift metabolic reprogramming, favoring aerobic glycolysis over oxidative phosphorylation (OxPhos), similar to the Warburg effect [44]. In pulmonary vascular fibroblasts, this metabolic shift increases the availability of metabolites that promote anabolic reactions, thereby promoting proliferation and inflammatory activation. Metabolic reprogramming toward glycolysis and the subsequent dominance of this pathway in fibroblasts are key drivers of remodeling and vascular inflammation in PAH [45].

The research by Chen et al. confirms the hypotheses regarding the impact of glycolytic reprogramming during PAH and expands on the role of the glycolytic protein alpha 1 subunit of prolyl 4-hydroxylase (P4HA1). Studies have shown a significant upregulation of P4HA1 in human pulmonary artery endothelial cells (HPAECs) under hypoxic conditions. Knockdown of P4HA1 effectively inhibited hypoxia-induced glycolytic reprogramming and reduced HPAEC migration capacity. In vitro studies confirm that suppression of P4HA1 protein in the endothelium reduces pulmonary vascular remodeling and improves right ventricular function in a mouse model. The study indicated P4HA1 as an important regulator of pulmonary hypertension pathogenesis and a potential target [46].

Critically, metabolic changes also affect platelets, which exhibit increased basal glycolysis and a lower glycolytic reserve than those of healthy individuals [38]. Platelets from PAH patients demonstrate a shift toward aerobic glycolysis characterized by increased extracellular acidification rates and augmented lactate production. This glycolytic shift provides rapid ATP generation necessary for sustained activation, degranulation, and secretion of pro-inflammatory mediators. McDowell et al. demonstrated that platelet glycolytic metabolism correlates with hemodynamic severity in Group 1 PH (PAH) patients [38]. Nguyen et al. further demonstrated that platelets in PAH exhibit an increased mitochondrial reserve capacity driven by enhanced fatty acid oxidation (FAO). Notably, this reserve correlates directly with mean pulmonary arterial pressure and pulmonary vascular resistance. Such findings underscore a unique metabolic flexibility: platelets concurrently maintain high mitochondrial efficiency despite a distinct shift toward aerobic glycolysis. This dual-track metabolic profile represents an adaptive response to chronic hemodynamic stress [39]. In the broader context of PAH, lactate accumulation has been shown to act as a signaling molecule that promotes SMC proliferation and stabilizes HIF-1α in vascular cells, thereby perpetuating a hypoxic, pro-remodeling microenvironment [43,45,46].

### 7.2. Mitochondrial Metabolic Rewiring

Remodeling mitochondrial metabolism drives a shift from traditional oxidative phosphorylation to less characteristic processes as the primary means of energy production in cells. The classic Warburg effect underpins metabolic reprogramming; however, the literature emphasizes glutaminolysis and FAO as the primary energy sources under conditions of metabolic stress. According to Culley et al., glutaminolysis is an anaplerotic reaction that replenishes Krebs cycle intermediates. This reaction allows rapidly proliferating pulmonary vascular cells to obtain the biomass necessary for their growth. Metabolic reprogramming, characterized by an increase in FAO to cardiomyocyte energy production, reaches 60–90% and may lead to lipotoxicity, resulting in increasingly advanced mitochondrial dysfunction [40].

The study by Yuan et al. emphasizes that mitochondrial dysfunction plays a significant role in the pathogenesis of PAH. In addition to energy metabolism disorders, mitochondrial dysfunction also involves damage to mitochondrial DNA (mtDNA), defects in the electron transport chain, protein homeostasis imbalance, defects in mitochondrial biogenesis, and abnormalities in mitochondrial dynamics and autophagy. The authors highlight the therapeutic potential of mitochondrial dysfunction-targeting treatments in PAH [41].

### 7.3. Fatty Acid Oxidation and Glutaminolysis

Beyond glycolysis and mitochondrial OxPhos, emerging evidence suggests that alterations in FAO and glutaminolysis contribute to the metabolic reprogramming of vascular cells in PAH. Nguyen et al. directly demonstrated that the increased mitochondrial reserve capacity observed in PAH platelets is dependent on enhanced FAO; inhibition of FAO with etomoxir abolished this increased reserve capacity, confirming that PAH platelets actively upregulate fatty acid catabolism as a bioenergetic adaptation [39]. This finding parallels observations in other cell types relevant to PAH (PASMCs, right ventricular cardiomyocytes, and macrophages), where alterations in FAO are associated with lipid accumulation, membrane remodeling, and shifts in substrate utilization [42,43]. Similarly, glutaminolysis—the metabolic conversion of glutamine to α-ketoglutarate to support the tricarboxylic acid (TCA) cycle—is a recognized feature of activated immune cells and proliferating vascular cells in PAH, supporting biosynthetic demands and redox homeostasis through glutathione production [42,43]. While the upregulation of glutaminolysis in PAH platelets remains to be definitively characterized through future metabolomic studies, the increased mitochondrial reserve capacity [39] and metabolic parallels between platelets and immune cells suggest that such anaplerotic pathways are likely engaged to sustain the metabolically demanding hyperactivated state.

### 7.4. Bioenergetics and Signaling Molecules

The connection between mitochondrial dysfunction and platelet bioenergetics in PAH represents a critical area of investigation. As discussed in Section 3, excessive ROS production under hypoxic conditions is a hallmark of PAH. Nguyen et al. demonstrated that platelets from PAH patients exhibit increased mitochondrial oxygen consumption rates and elevated spare respiratory capacity compared to healthy controls, suggesting that platelet mitochondria undergo adaptive reprogramming to cope with the sustained metabolic demands of chronic activation [39]. Elevated mitochondrial ROS (mtROS) generation in the context of PAH contributes to platelet activation through oxidation of signaling proteins and calcium mobilization; at excessive levels, oxidative damage to membrane lipids and proteins promotes the release of pro-inflammatory extracellular vesicles (EVs) [36,39]. Furthermore, mitochondrial dysfunction in platelets is linked to opening of the mitochondrial permeability transition pore (mPTP), which triggers phosphatidylserine externalization (a critical step in generating procoagulant platelet surfaces and PDMPs) [36].

This mechanism directly connects platelet mitochondrial dysfunction to the hypercoagulable state and vesicle-mediated signaling observed in PAH. Hua et al. demonstrated that megakaryocytes increase thrombopoiesis under conditions of hyperoxia or oxidative stress, and that mtROS promote their maturation and platelet production [47]. These results suggest that targeting platelet mitochondria therapeutically may slow the progression of PAH by simultaneously addressing platelet hyperactivation, vesicle release, and procoagulant activity.

In this context, ROS in platelets are not merely byproducts of oxidative stress but rather serve as precise signals that stimulate the release of secondary signaling molecules. An example is the activation of proliferative pathways by HIF-1α, leading to the proliferation of PASMCs and ECs. This way, HIF-1α indirectly influences vascular remodeling. ROS is also present in ion channels, which induce sustained, severe contraction of pulmonary vessels by increasing calcium influx into the cell. Furthermore, excessive ROS production activates transcription factors such as NF-κB, which promote the release of pro-inflammatory cytokines, thereby exacerbating vascular damage [48]. Sekar et al. also highlight the role of ROS in EV formation [49]. Platelet-derived EVs thus induce endothelial dysfunction and the prothrombotic and inflammatory state observed in PAH [50].

The scientific literature clearly highlights the role of platelets in the development and progression of PAH beyond oxidative signaling. According to Kazimierczyk et al., platelets release a wide variety of chemokines in PAH; however, inflammatory modulators such as IL-6 and interleukin-1β (IL-1β) play a direct role in the disease’s development. A platelet-derived cytokine, soluble tumor necrosis factor-like weak inducer of apoptosis (sTWEAK), plays a role in numerous biological processes related to tissue damage and repair, including apoptosis, cell proliferation, and angiogenesis. The authors clearly emphasize that microparticles derived from platelets are increasingly recognized as a marker of progressive PAH [51]. The current literature also underscores the critical interactions between platelets and neutrophils in the process of immunothrombosis (NET formation). In this process, activated platelets stimulate neutrophils to release extracellular neutrophil traps, which serve as scaffolds for subsequent platelet aggregation and fibrin accumulation [52].

### 7.5. Metabolic Stress-Induced Vesiculation

EVs are spherical structures surrounded by a double phospholipid membrane and are a heterogeneous group of structures. While exosomes are of endosomal origin, the microparticles (microvesicles) primarily released by activated platelets in PAH are formed by the outward budding and shedding of the plasma membrane in response to various signals, including those associated with metabolic disorders. They transport chemokines, cytokines, lipids, and various forms of RNA, serving as carriers of molecules with significant biological activity. A growing body of research demonstrates that EVs may play a negative role in the development of pulmonary hypertension by transmitting molecular signals that promote the progression of this disease [53]. The release of EVs from platelets is promoted by metabolic stress, including mitochondrial dysfunction and oxidative damage, which drive phosphatidylserine externalization and vesicle shedding, effectively connecting the bioenergetic alterations in platelets to their paracrine signaling capacity [36,53].

It has been demonstrated that individuals with PAH exhibit elevated levels of EVs derived from platelets, ECs, and erythrocytes, which have a proangiogenic effect on PAECs. Furthermore, platelet-derived vesicles in patients with PAH can evade lysosomal degradation in PAECs [39]. EVs can promote the transcription and translation of numerous proangiogenic proteins and their receptors in HPAECs. It has been observed that they increase the expression of mRNA for VEGF-A, its receptor, as well as for placental growth factor (PGF) and fibroblast growth factor-2 (FGF2) in HPAECs [54]. Kosanovic et al. reported elevated levels of T-lymphocyte-derived EVs in PAH patients, further confirming the inflammatory nature of the disease, in which immune cells accumulate in lung tissue and remodel pulmonary vessels. The authors also noted that the EV pattern varied across disease subgroups [55].

Elevated EV concentrations are also observed in patients with PAH developing as a complication of systemic lupus erythematosus (SLE-PAH). Ding et al. observed an increased number of vesicles derived from leukocytes, erythrocytes, platelets, and endothelial cells. The results analysis revealed that specific populations of circulating EVs may promote the development of hypercoagulability and exacerbate the course of SLE-PAH [56]. These data are consistent with the findings of Hasse et al., who experimentally demonstrated increased platelet marker activation in patients with SLE and elevated levels of phosphatidylserine-positive REV (PS^+^REV). The results conclusively demonstrate an intensified vascular inflammatory process and increased susceptibility to cardiovascular complications secondary to SLE-associated PAH [57].

### 7.6. Pro-Inflammatory Mediators from Platelets

One of the key mechanisms initiating PAH is pulmonary vascular endothelial dysfunction, in which disorders of the platelet-dependent serotonin system play a significant role. In pediatric cohort studies of patients with congenital heart defects complicated by pulmonary hypertension, serum and platelet serotonin concentrations, as well as serotonin transporter (SERT) expression on platelets, were evaluated. Enhanced SERT activity in platelets was observed in the PAH group, accompanied by a simultaneous tendency toward increased plasma serotonin levels and decreased platelet serotonin content. Available evidence suggests impaired SERT function despite its increased expression. At the same time, studies have shown that SERT participates in PASMC proliferation, promoting vascular remodeling and PAH progression in children with congenital heart defects [58]. This correlation was also confirmed by Mindubayeva et al. in a study of children who underwent surgical correction of heart defects. In the preoperative period, elevated platelet counts and increased serotonin levels were observed, whereas postoperative measurements showed a significant decrease in platelet and serotonin content. Collectively, these data indicate that a close relationship exists between platelet activity, serotonin metabolism, and the development of PAH in congenital heart defects [59].

Platelet-Derived Growth Factor (PDGF) is another platelet-derived mediator involved in remodeling pulmonary vessels in PAH. PDGF transmits signals via tyrosine kinase receptors, particularly the ß-PDGF receptor (ß-PDGFR), which is considered the primary regulator of vascular smooth muscle cell (VSMC) proliferation. PAH patients have been shown to have increased expression and phosphorylation of ß-PDGFR, confirming the activation of this signaling pathway in the pathogenesis of the disease. ß-PDGFR activation plays a crucial role in remodeling the pulmonary artery wall, particularly under hypoxic conditions. Data from in vivo models suggest that selective disruption of PDGF signaling reduces the development of pulmonary hypertension, inhibits vascular changes, and decreases secondary remodeling of the right ventricle, supporting its pathogenic role [60]. This is further supported by studies demonstrating elevated levels of PDGF-β in IPAH in macrophages derived from circulating monocytes, which are an important source of PDGF extravascularly, thereby exacerbating the local inflammatory response and vascular remodeling [61].

The significance of PDGF signaling is supported by pharmacological studies showing that PDGF receptor inhibitors reverse vascular changes in experimental models of PAH. Although tyrosine kinase inhibitors (TKIs), including Imatinib, have demonstrated beneficial hemodynamic effects, their limited specificity and adverse effects significantly reduce the clinical usefulness of this strategy. Ongoing research is focused on developing new, more selective therapeutic approaches that target PDGF signaling [62].

## 8. Vascular Remodeling

**Figure 2:**
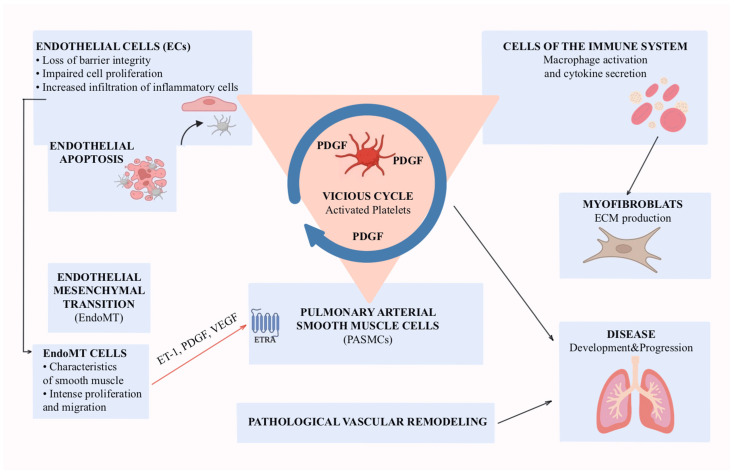
Vascular remodeling in PAH.

The consequences of complex endothelial dysfunction in PAH, including loss of barrier integrity, impaired cell proliferation, and increased infiltration of inflammatory cells, lead to pathological vascular remodeling. Inflammation and cytokine secretion, together with endothelial apoptosis, disrupt the balance between damage and repair, leading to the formation of complex, occlusive arterial lesions during the development and progression of the disease (Figure 2) [63,64].

The immunometabolic reprogramming of platelets described in Section 7 directly contributes to these structural changes. Glycolytically reprogrammed platelets produce excess lactate, which in the broader pulmonary vascular environment stabilizes HIF-1α in PASMCs and fibroblasts, promoting their proliferation and resistance to apoptosis [42,43,45]. Platelet-derived EVs transfer proangiogenic cargo (VEGF-A, FGF2, PGF) to ECs, stimulating aberrant angiogenesis [54]. Concurrently, the sustained release of PDGF and serotonin from activated platelets drives SMC hypertrophy and medial thickening [58,59,60,61,62]. The platelet-derived inflammatory mediators recruit monocytes and macrophages, which, in turn, amplify the remodeling cascade by producing additional growth factors [14,17,27]. Thus, the metabolically reprogrammed platelet acts as a nexus connecting immunothrombosis to structural vascular remodeling in PAH.

Chronic stress and inflammatory factors can induce endothelial–mesenchymal transition (EndoMT) in ECs, in which PAECs transform into cells with smooth muscle cell characteristics, capable of intense proliferation and migration [65]. As a result of this process, EndoMT cells begin to release stimulatory factors such as ET-1, PDGF, and VEGF, which promote the proliferation and migration of PASMCs, leading to thickening of the vessel walls and obstruction of distal pulmonary capillaries. Additionally, ET-1 further activates the endothelin A receptor (ETRA), resulting in sustained vasoconstriction and smooth muscle proliferation [9,65]. PAECs can transform into fibroblasts, which, under the influence of factors such as ET-1, IL-6, hypoxia, inflammatory cytokines, and growth factors, differentiate into highly proliferative and contractile myofibroblasts that produce ECM and contribute to vascular remodeling [65,66]. These findings highlight the important role of EndoMT in the pathogenesis of PAH and underscore the need for further research into the potential reversal of this process in pathological conditions [67].

Recent studies suggest that macrophages coordinate interactions between pro-inflammatory and anti-inflammatory mediators, as well as between SMCs and fibroblasts. The activation of macrophages in PAH models triggers changes in multiple signaling pathways and the production of various cytokines. Preliminary preclinical studies have identified potential therapeutic targets in macrophages, making them a promising target for the treatment of PAH, although further research is needed [68]. Current PAH therapies primarily focus on dilating pulmonary vessels without addressing pathological vascular remodeling. Accordingly, research is focused on developing novel therapies directed at important pathogenic mechanisms, including bone morphogenetic protein signaling, tyrosine kinase receptors, serotonin metabolism, angiogenesis, extracellular matrix remodeling, estrogen signaling, and epigenetic regulation [68,69].

The case report by Erickson-Keith describing a patient with PAH perfectly illustrates how immunometabolic reprogramming influences vascular remodeling. The case describes a patient with PAH in whom metabolic reprogramming was observed, characterized by a shift from oxidative phosphorylation to enhanced anaerobic glycolysis. This process provides the necessary energy and precursors for intense cell proliferation in PAH. These changes directly exacerbate inflammation, stiffen the extracellular matrix, and thicken the vascular wall. This results in remodeling, which leads to a narrowing of the vessel lumen and permanent circulatory dysfunction in the course of PAH [70].

## 9. Clinical Relevance

### 9.1. Laboratory Parameters of Platelets

Platelets are now recognized as key elements actively involved in the immune-inflammatory response, which may contribute to the pathogenesis and influence the prognosis of PAH [71]. Although hemodynamic parameters provide the evidentiary basis for determining progression and prognosis in PAH, there is a need for non-invasive biomarkers. Notwithstanding the identification of numerous potential prognostic indicators in PAH, a highly sensitive and specific biomarker has yet to be identified [25].

Red blood cell distribution width (RDW) is frequently mentioned in the literature as a prognostic indicator in PAH. However, RDW is a systemic marker reflecting chronic inflammation, microcirculatory damage, and hypoxia-induced erythropoiesis rather than a direct marker of platelet function. The extant literature consistently demonstrates that platelet count is a quantitative biomarker of PAH [72]. Corroborating evidence, confirming reduced platelet counts in patients with PAH, was also presented in studies by Fernandes et al. [71]. The existing literature reports that increased RDW and decreased platelet count are consequences of increasing hypoxia, which promotes preferential stimulation of erythropoiesis at the expense of megakaryopoiesis, thereby disrupting the balance of hematopoietic lines in PAH [72]. A comprehensive analysis of published data suggests a positive correlation between platelet count and the risk of developing PAH [73].

MPV reflects platelet size and biological activity and is associated with many cardiovascular diseases. Larger platelets exhibit increased metabolic and enzymatic activity, resulting in a higher prothrombotic and proinflammatory potential. Divergences in MPV profiles between PAH patients and normotensive individuals may reflect varying degrees of platelet activation, leading to the release of distinct inflammatory mediators and, consequently, affecting pulmonary vascular remodeling [71]. PDW reflects differences in platelet size, reflecting heterogeneity resulting from morphological changes, such as pseudopod formation. A prominent elevation in PDW values may be associated with increased prothrombotic activity and chronic activation of the coagulation system in the pulmonary circulation, exacerbated by microtrauma to the endothelium and hypoxia. However, the exact mechanism of this phenomenon remains unclear [74].

MPV and PDW are platelet activation indices currently considered potential risk factors for cardiovascular disease. However, it must be strongly emphasized that these are highly non-specific markers of systemic inflammation observed across a wide spectrum of cardiovascular conditions. While accumulating evidence suggests that both MPV and PDW are elevated in patients with IPAH, which may partly reflect disease severity [75], their diagnostic utility in isolation remains limited. Comparable findings were reported by Awad et al., who observed increased MPV, PDW, and plateletcrit (PCT) values in children with PAH associated with congenital heart disease (PAH-CHD), which correlate with PAH severity, right ventricular diameter, and mean pulmonary artery pressure [25]. Markedly elevated MPV is also observed in patients with chronic obstructive pulmonary disease (COPD) who have PAH secondary to COPD, with MPV correlating positively with the severity of PAH [76].

Regarding the quantitative platelet indicator, scientific reports describe it in patients with PAH. Mirad et al. describe thrombocytopenia as a significant challenge during acute decompensation of PAH, which researchers partly attribute to platelet aggregation in the pulmonary circulation. Scanning Electron Microscopy (SEM) studies have revealed capillaries filled with platelet aggregates, which explains the decrease in their number in PAH during acute decompensation [22]. Building on these findings, Le et al. observed that reduced platelet count is an independent risk factor for death in IPAH, which can be used to assess disease severity and its association with acute decompensation [77].

Thrombocytopenia poses a significant clinical challenge in the treatment of PAH with prostacyklin analogs. In response to this problem, Grześk et al. developed a proprietary therapeutic regimen in line with current hematology guidelines. The proposed model provides decision support for clinicians, allowing them to modify treatment depending on the current platelet count and the severity of hemorrhagic symptoms [78].

In terms of morphological differences, Balko et al. observed a marked increase in the number of pulmonary megakaryocytes and changes in their structure in patients with IPAH. This discovery may contribute to a better understanding of the mechanisms of vasoconstriction, thrombosis, and vascular remodeling in IPAH, as evidence suggests that megakaryocyte and platelet products may reflect these processes [79]. Hua et al. demonstrated that megakaryocytes increase thrombopoiesis under conditions of hyperoxia or oxidative stress, and that mitochondrial reactive oxygen species (mtROS) promote their maturation and platelet production. These results suggest the potential of targeting platelet mitochondria with therapy to slow the progression of PAH and improve therapeutic response [47]. To synthesize the extensive data on morphological and quantitative platelet changes in PAH, Table 1 summarizes key laboratory platelet parameters, their observed trends, and their clinical significance.

### 9.2. Pharmacological Control of Platelet Function and Hemostasis

When analyzing platelet function in PAH, it is important to clearly separate disease-driven platelet abnormalities from secondary changes induced by targeted therapies. Therapeutic prostacyclin analogs (epoprostenol, treprostinil, iloprost) have a significant vasodilatory effect in PAH and also directly affect hemostasis by inhibiting platelet activity. Gąsecka et al. assessed platelet function in patients with PAH treated with PGI_2_ analogs using multi-electrode aggregometry, the T-TAS system (PL microchip) for analysis of clot formation, and flow cytometry to determine the EV subpopulation. A significant reduction in platelet reactivity, decreased EV release, and impaired clot formation were observed compared with patients not treated with prostacyclin analogs [81,82]. In turn, Siniarki et al. confirmed reduced platelet aggregation in patients undergoing pharmacological therapy but did not observe significant differences in clot development and lysis parameters compared to the group not receiving PGI_2_ analogs [83].

PDE5 inhibitors are a pivotal element of pharmacotherapy and also influence platelet function regulation. Physiologically, the endothelium maintains platelet activity by releasing anti-aggregatory factors, including NO, which activates GC and increases intracellular cGMP levels. Activation of the cGMP-protein kinase G pathway leads to inhibition of platelet activation. The level of cGMP is precisely controlled by the balance between its synthesis and degradation catalyzed by phosphodiesterase, among which PDE5 exhibits high specificity for cGMP and is intensively expressed in tissues. In clinical practice, PDE5i drugs are the first-line therapy for PAH, and their ability to inhibit platelet aggregation suggests potential anticoagulant benefits, both in monotherapy and in combination, particularly in the context of cardiovascular disease and in limiting ischemia [84,85,86,87]. According to Grześk et al., modulating GC activity is currently one of the most promising approaches for pharmacotherapy development [88]. Therefore, any ex vivo assessment of platelet function or global hemostasis in treated PAH patients must account for the potent antiplatelet effects of these specific therapies.

The clinical utility of anticoagulant therapy in PAH remains a topic of debate in the research literature. The foundational premise for this therapy was derived from autopsy studies, which showed a high incidence of in situ thrombosis in the pulmonary vessels of patients with PAH. Subsequent observations suggested that PAH is associated with dysregulation of the coagulation system and anticoagulant mechanism, promoting the development of a prothrombotic phenotype. Post mortem examinations, biomarker studies, and translational models clearly indicate an imbalance between coagulation and fibrinolysis in PAH, requiring anticoagulant therapy [89].

A meta-analysis by Khan et al. showed that anticoagulant therapy may improve survival in patients with IPAH but is associated with increased mortality in PAH associated with systemic sclerosis. The therapeutic efficacy of anticoagulant therapy includes reducing thrombosis and hypercoagulability, as well as modulating the proliferative effects of thrombin. However, its use requires an individualized risk assessment and disease phenotype [90].

Contemporary anticoagulation regimens in PAH remain heterogeneous, partly due to limited and inconclusive data on global hemostasis assessment tests. Lu et al. conducted a seminal analysis of thromboelastography (TEG) in patients with PAH treated with vasodilators. The aforementioned study did not reveal any significant abnormalities in coagulation or fibrinolysis kinetics compared to healthy individuals. Most TEG parameters were comparable between groups, except for LY30, which indicated lower fibrinolytic activity in patients with PAH. It is essential to note that all LY30 values fell within the reference range, indicating that global hemostasis was preserved in treated patients [91].

Different results were presented by Virgkou et al., who analyzed patients with newly diagnosed PAH who were not receiving vasodilator therapy. The authors found prolonged clotting time and delayed clot formation, suggesting significant hemostatic disorders in the early stages of the disease [15]. The absence of targeted therapy for pulmonary vessels may have significantly influenced the observed abnormalities. This group’s subsequent research confirmed that patients with PAH exhibit reduced platelet aggregation and disaggregation, impaired clot initiation and propagation, and reduced thrombin generation capacity, indicating complex hemostatic dysregulation in untreated patients [15,16]. The evaluation of platelet function and hemostatic dysregulation in PAH requires a multifaceted approach. Various advanced analytical techniques have been employed across different studies to capture the complexity of these processes. Table 2 summarizes the key hemostasis assessment methods mentioned in the reviewed literature, highlighting their specific applications and primary findings in the context of PAH.

### 9.3. Cornerstones of PAH Therapy and Ex Vivo Assays

The cornerstone of therapy in PAH comprises prostacyclin analogs [78]. The prostacyclin metabolic pathway plays a significant role in the pathophysiology of PAH; patients have been shown to exhibit reduced prostacyclin synthase expression, as evidenced by decreased concentrations of its metabolites in urine. In clinical practice, pharmacological agents targeting the prostacyclin pathway are used, with therapeutic effects based on vasodilation, inhibition of platelet aggregation, and antiproliferative activity [92]. Although multiple prostacyclin pathway-based therapies are available, there is still a lack of direct comparative studies evaluating their efficacy and safety [A], and one of the major challenges encountered by clinicians during pharmacotherapy is thrombocytopenia [78].

Another critical aspect of prostacyclin analog therapy is the inhibition of platelet function, which complicates reliable interpretation of ex vivo assays. These agents, through potent suppression of platelet activity, delay thrombus formation and reduce clot size, which directly contributes to decreased platelet reactivity compared with control groups [81]. In this context, in patients treated with prostacyclin analogs, aggregometry results demonstrate impaired clot formation, leading to a misleading assumption of hemostatic imbalance that is, in fact, primarily driven by pharmacological effects [81].

The second cornerstone of PAH therapy consists of phosphodiesterase type 5 inhibitors (PDE5i), which, through modulation of the NO/cGMP/PKG signaling pathway in platelets, effectively inhibit their activation. As described in Section 9.2, Pharmacological Control of Platelet Function and Hemostasis, the ability of PDE5i to inhibit platelet aggregation confers potential antithrombotic benefits [84,85,86,87], while simultaneously complicating the reliable interpretation of ex vivo assays.

## 10. Limitations

We acknowledge that this narrative review does not provide a systematic or quantitative synthesis of the literature. The selection of studies may be influenced by publication bias and by limited access to unpublished data or the full texts of some studies. Additionally, while our primary search targeted the last decade, the inclusion of foundational studies and prior reviews means that the evidence base is not strictly restricted to 2015–2025. Nevertheless, this approach allows us to extend the literature review, identify the main trends and concepts in the area under study, and identify gaps in the literature that may be the subject of future research.

## 11. Future Directions

Despite advances in understanding platelet immunometabolism in PAH, the transition from molecular discoveries to routine clinical practice still poses a challenge. Future research should go beyond the standard correlation of immunometabolic changes with disease progression, focusing on whether platelet changes are a primary element of pathogenesis or merely a secondary response to systemic inflammation associated with PAH.

A key limitation of current reports is the lack of standardized protocols for platelet functional analysis and isolation, which prevents direct comparisons of results across research centers and undermines their reliability. Therefore, rigorous methodological standardization and validation of advanced hemostasis tests, such as TEG^®^ or Total Thrombus-Formation Analysis System (T-TAS^®^), are essential in multicenter prospective clinical trials. Only when consistent parameters are obtained will it be possible to use them objectively in personalizing antiplatelet therapy. From a practical perspective, the primary beneficiaries of this research will be clinicians, who will gain precise tools for patient phenotyping to guide targeted PAH therapy, and laboratory specialists, for whom platelet parameters may serve as sensitive biomarkers of pulmonary vascular status.

Future studies should also specifically address the metabolic profiling of PAH platelets using metabolomics and lipidomics approaches, to definitively characterize the contributions of FAO, glutaminolysis, and other anaplerotic pathways to the platelet hyperactivation phenotype. Additionally, the role of platelets in stimulating NET formation and the reciprocal effects of NETs on platelet metabolism warrant further investigation as potential therapeutic targets.

Current scientific reports should be treated with criticism. Without a critical assessment of the permanence of platelet changes and their reactivity to standard PAH pharmacotherapy, platelet immunometabolism will remain only an attractive theoretical concept. The ultimate success of this field will depend on demonstrating that modulation of platelet function inhibits pulmonary vascular remodeling rather than simply improving isolated laboratory parameters.

## 12. Conclusions

Analysis of data collected over the last decades confirms that platelets in PAH have evolved from passive morphotic elements involved in thrombosis to active mediators of inflammation and pulmonary vascular remodeling. A review of the literature in this area points to the key role of platelets in immunothrombosis: platelets serve as a link between endothelial dysfunction and the systemic inflammatory response, acting not only through aggregate formation but also through the release of pro-inflammatory mediators and growth factors (PDGF, serotonin) that recruit and active immune cells.

Furthermore, platelet interaction with leukocytes, including neutrophil NET formation, not only promotes chronic inflammation but also serves as a sensitive biomarker of cellular activation in PAH. Moreover, the immunometabolic theory of platelet metabolic reprogramming toward aerobic glycolysis (Warburg effect), coupled with mitochondrial dysfunction and likely alterations in FAO and glutaminolysis, are integral features of PAH pathogenesis; increased lactate production, mediator release, and EVs directly drive pathological SMC proliferation and pulmonary vascular remodeling.

A review of the literature also points to a hemostatic paradox (the platelet “paradox”), in which chronic platelet activation in a hypoxic, oxidation-stress environment leads to depletion of hemostatic potential. This manifests as reduced reactivity while maintaining a prothrombotic state, requiring the use of more precise diagnostic tools such as TEG or T-TAS.

The diagnostic value of routine morphological parameters such as MPV and PDW, despite their non-specificity, reflects the degree of platelet metabolic activation and correlates with the severity of PAH. Nevertheless, advanced global hemostasis assessment tests offer hope for personalized supportive therapy and better monitoring of the effect of targeted drugs on the coagulation system.

## Figures and Tables

**Table 1:** Summary of key platelet laboratory parameters and their alterations in PAH.

Parameter	Alteration in PAH	Clinical Significance	References
Platelet Count	↓↓	Independent risk factor for mortality in IPAH; correlates with acute decompensation	[71,72,77]
MPV non-specific marker	↑ *	Correlates with PAH severity and mPAP in IPAH and PAH-CHD; direction inconsistent across populations	[25,71,75]
PDW non-specific marker	↑↑	Reflects platelet heterogeneity and chronic activation; elevated in IPAH and PAH-CHD	[25,63]
PCT	↑↑↑	Correlates with PAH severity, mPAP, and RV diameter in PAH-CHD	[25]
RDW	↑	Systemic marker; correlates with sPAP; reflects hypoxia-induced erythropoiesis	[72]
P-selectin	↑	Marker of platelet activation and endothelial damage	[16]
Serotonin (plasma)	↑ *	Supports serotonin hypothesis; SERT upregulation promotes PASMC proliferation; distinction between plasma and local synthesis is important	[16,19,58]
TXA	↑↑	Marker of persistent prothrombotic signaling	[16,20,80]
PDMPs	↑↑	Biomarker of pathological platelet activation; PDMP membranes exhibit 50–100× greater specific procoagulant activity than activated platelets (general property)	[35,36]

The Scale column represents the estimated magnitude of change: ↑ = mild (<15% change or not quantified), ↑↑ or ↓↓ = moderate (15–50% change or ~2–3-fold), ↑↑↑ = marked (>50% change). An asterisk (*) indicates inconsistent direction across studies or contested evidence. Note: [76] Mohamed 2019 (MPV in COPD-associated PH) and [74] Wang 2021 (PDW in CLD-PH) were excluded from this table as they pertain to WHO Group 3 PH rather than Group 1 PAH; [73] Li 2024 (Mendelian randomization) was excluded as it examines platelet count as a risk factor for developing PAH rather than measuring platelet count alterations in PAH patients. These studies are discussed in the text. Abbreviations: MPV, mean platelet volume; PDW, platelet distribution width; PCT, plateletcrit; RDW, red blood cell distribution width; TXA, thromboxane; PDMPs, platelet-derived microparticles; mPAP, mean pulmonary artery pressure; sPAP, systolic pulmonary artery pressure; IPAH, idiopathic pulmonary arterial hypertension; PAH-CHD, PAH associated with congenital heart disease; PASMC, pulmonary artery smooth muscle cell; SERT, serotonin transporter; RV, right ventricle.

**Table 2:** Overview of hemostasis assessment methods and their key findings in PAH.

Test/Method	Key Findings in PAH	References
LTA (EPI, ADP)	Reduced platelet aggregation and aggregate stability	[15,16,18]
ROTEM	Prolonged clot initiation; delayed fibrinolysis	[18]
TEG	Preserved hemostasis in treated patients; reduced LY30	[91]
T-TAS	Impaired clot formation in prostacyclin-treated patients	[81]
Thrombin generation	Reduced capacity in untreated PAH	[15,16]

Note that T-TAS, multi-electrode aggregometry, and flow cytometry findings reflect the pharmacological effect of prostacyclin therapy rather than disease-intrinsic hemostatic alterations. The thrombin generation results are derived from newly diagnosed, untreated PAH patients; however, conflicting data exist from other studies using similar methodology. Abbreviations: LTA, light transmission aggregometry; EPI, epinephrine; ADP, adenosine diphosphate; ROTEM, rotational thromboelastometry; TEG, thromboelastography; LY30, percent lysis at 30 min; T-TAS, Total Thrombus-Formation Analysis System; PAH, pulmonary arterial hypertension.

## Data Availability

No new data were created or analyzed in this study. Data sharing is not applicable to this article.

## References

[1] Wang A., Pan Q., Zhang J., Gong S., Zhang F., Liang N., Yang Y., Jiang Z. (2025). Role of inflammation in endothelial responses in Pulmonary Hypertension. Biomed. Pharmacother..

[2] Hu Y., Chi L., Kuebler W.M., Goldenberg N.M. (2020). Perivascular Inflammation in Pulmonary Arterial Hypertension. Cells.

[3] Guo Y., Gao Z., Zhang R., Long H., Zhang M., Liu L., An Z., Shi Y., Cui Y., Jia Y. (2025). Neutrophil-Endothelium Interaction Mediated by S100A9 Promotes Pulmonary Vascular Remodeling During Pulmonary Hypertension. Adv. Sci..

[4] Song Y., Jia H., Ma Q., Zhang L., Lai X., Wang Y. (2024). The causes of pulmonary hypertension and the benefits of aerobic exercise for pulmonary hypertension from an integrated perspective. Front. Physiol..

[5] Sydykov A., Mamazhakypov A., Maripov A., Kosanovic D., Weissmann N., Ghofrani H.A., Sarybaev A.S., Schermuly R.T. (2021). Pulmonary Hypertension in Acute and Chronic High Altitude Maladaptation Disorders. Int. J. Environ. Res. Public Health.

[6] Kurakula K., Smolders V.F.E.D., Tura-Ceide O., Jukema J.W., Quax P.H.A., Goumans M.-J. (2021). Endothelial Dysfunction in Pulmonary Hypertension: Cause or Consequence?. Biomedicines.

[7] Correale M., Mercurio V., Bevere E.M.L., Pezzuto B., Tricarico L., Attanasio U., Raucci A., Ferrara A.L., Loffredo S., Puteo C. (2025). Pathophysiology of Pulmonary Arterial Hypertension: Focus on Vascular Endothelium as a Potential Therapeutic Target. Int. J. Mol. Sci..

[8] Nowaczyk A., Kowalska M., Nowaczyk J., Grześk G. (2021). Carbon Monoxide and Nitric Oxide as Examples of the Youngest Class of Transmitters. Int. J. Mol. Sci..

[9] Otani N., Tomoe T., Kawabe A., Sugiyama T., Horie Y., Sugimura H., Yasu T., Nakamoto T. (2022). Recent Advances in the Treatment of Pulmonary Arterial Hypertension. Pharmaceuticals.

[10] Grześk G., Witczyńska A., Grześk G., Witczyńska A., Węglarz M., Wołowiec Ł., Nowaczyk J., Grześk E., Nowaczyk A. (2023). Guanylyl Cyclase Activators-Promising Therapeutic Option in the Pharmacotherapy of Heart Failure and Pulmonary Hypertension. Molecules.

[11] Fellows A.L., Quigley K., Leung V., Ainscough A.J., Wilkins M.R., Barnett H., Miller D., Mayr M., Wojciak-Stothard B. (2025). Engineered pulmonary artery tissues for measuring contractility, drug testing and disease modelling. Br. J. Pharmacol..

[12] Truss N.J., Warner T.D. (2011). Gasotransmitters and platelets. Pharmacol. Ther..

[13] Aytekin M., Aulak K.S., Haserodt S., Chakravarti R., Cody J., Minai O.A., Dweik R.A. (2012). Abnormal platelet aggregation in idiopathic pulmonary arterial hypertension: Role of nitric oxide. Am. J. Physiol. Lung Cell. Mol. Physiol..

[14] Lannan K.L., Phipps R.P., White R.J. (2014). Thrombosis, platelets, microparticles and PAH: More than a clot. Drug Discov. Today.

[15] Vrigkou E., Tsangaris I., Bonovas S., Kopterides P., Kyriakou E., Konstantonis D., Pappas A., Anthi A., Gialeraki A., Orfanos S.E. (2019). Platelet and coagulation disorders in newly diagnosed patients with pulmonary arterial hypertension. Platelets.

[16] Vrigkou E., Tsantes A.E., Kopterides P., Orfanos S.E., Armaganidis A., Maratou E., Rapti E., Pappas A., Tsantes A.G., Tsangaris I. (2020). Coagulation Profiles of Pulmonary Arterial Hypertension Patients, Assessed by Non-Conventional Hemostatic Tests and Markers of Platelet Activation and Endothelial Dysfunction. Diagnostics.

[17] Cullivan S., Murphy C.A., Weiss L., Comer S.P., Kevane B., McCullagh B., Maguire P.B., Ní Ainle F., Gaine S.P. (2021). Platelets, extracellular vesicles and coagulation in pulmonary arterial hypertension. Pulm. Circ..

[18] Vrigkou E., Tsantes A., Vrigkou E., Tsantes A., Konstantonis D., Rapti E., Maratou E., Pappas A., Halvatsiotis P., Tsangaris I. (2022). Platelet, Fibrinolytic and Other Coagulation Abnormalities in Newly-Diagnosed Patients with Chronic Thromboembolic Pulmonary Hypertension. Diagnostics.

[19] MacLean M.M.R. (2018). The serotonin hypothesis in pulmonary hypertension revisited: Targets for novel therapies (2017 Grover Conference Series). Pulm. Circ..

[20] Mahajan C.N., Afolayan A.J., Eis A., Teng R.J., Konduri G.G. (2015). Altered prostanoid metabolism contributes to impaired angiogenesis in persistent pulmonary hypertension in a fetal lamb model. Pediatr. Res..

[21] Delaney C., Davizon-Castillo P., Allawzi A., Posey J., Gandjeva A., Neeves K., Tuder R.M., Di Paola J., Stenmark K.R., Nozik E.S. (2020). Platelet activation contributes to hypoxia-induced inflammation. Am. J. Physiol. Lung Cell. Mol. Physiol..

[22] Miard C., de Montpreville V.T., Bernaudin J.F., Adam J., Djediat C., Stephan F. (2024). Platelet aggregates in lung capillaries in severely decompensated pulmonary hypertension. Thorax.

[23] Sun L., Li H., Li X., Li Y., Liu M., Tian H., Liu J., Miao R., Zhang Y., Huang Q. (2025). Dysregulation of immune cells and platelet-monocyte aggregates in chronic thromboembolic pulmonary hypertension. Respir. Res..

[24] Sun L., Li H., Li Y., Li X., Tian H., Liu J., Liu M., Wang J., Huang Q., Zhang Z. (2025). Proteomic insights into platelet dysregulation and pathogenic mechanisms of chronic thromboembolic pulmonary hypertension. J. Transl. Med..

[25] Awad A., Elnemr S., Hodeib H., El Amrousy D. (2022). Platelet Activation Markers in Children with Pulmonary Arterial Hypertension Associated with Congenital Heart Disease. Pediatr. Cardiol..

[26] Vrigkou E., Tsantes A., Bonovas S., Anthi A., Konstantonis D., Pappas A., Mitrakou C., Orfanos S., Armaganidis A., Tsagkaris I. (2016). Assessment of platelet dysfunction in patients with pulmonary hypertension. Eur. Respir. J..

[27] Nicoleau S., Wojciak-Stothard B. (2020). Thrombosis: The Role of Platelets in Pulmonary Hypertension. SciMedJ..

[28] Simonneau G., Torbicki A., Dorfmüller P., Kim N. (2017). The pathophysiology of chronic thromboembolic pulmonary hypertension. Eur. Respir. Rev..

[29] Åberg M., Björklund E., Wikström G., Christersson C. (2022). Platelet-leukocyte aggregate formation and inflammation in patients with pulmonary arterial hypertension and CTEPH. Platelets.

[30] Gerrits A.J., Frelinger A.L., Michelson A.D. (2016). Whole Blood Analysis of Leukocyte-Platelet Aggregates. Curr. Protoc. Cytom..

[31] Farrell C.L., Jordan M., Posey J.N., Jordan K.R., Gandjeva A., Nozik E.S., Stenmark K.R., Tuder R.M., Graham B.B., Delaney C.A. (2025). Platelet-Macrophage Aggregates in Remodeled Vessels of Patients with Pulmonary Arterial Hypertension. Pulm. Circ..

[32] Harper R.L., Zhou X., Marciano D.P., Cao A., Wang L., Chen G., Adil M.S., Zhou W., Maguire P., Deivanayagam S. (2025). Altered maturation and activation state of circulating monocytes is associated with their enhanced recruitment in pulmonary arterial hypertension. Respir. Res..

[33] Yang D., Li Q., Yang F., Wang R., Jiang P., Wu J., Yang X., Huang Y., Liu Y., Wang S. (2025). Machine learning-based integration develops an immune-derived signature for diagnosing high-altitude pulmonary hypertension. Front. Med..

[34] Wu X., He Y., Chen Z., He Z., Yan Y., He Y., Wang G., Dong Y., Yang Y., Sun Y. (2023). Single-cell analysis of peripheral blood from high-altitude pulmonary hypertension patients identifies a distinct monocyte phenotype. Nat. Commun..

[35] Ogawa A., Matsubara H. (2020). Increased levels of platelet-derived microparticles in pulmonary hypertension. Thromb. Res..

[36] Guo J., Cui B., Zheng J., Yu C., Zheng X., Yi L., Zhang S., Wang K. (2024). Platelet-derived microparticles and their cargos: The past, present and future. Asian J. Pharm. Sci..

[37] Kailashiya J. (2018). Platelet-derived microparticles analysis: Techniques, challenges and recommendations. Anal. Biochem..

[38] McDowell R.E., Aulak K.S., Almoushref A., Melillo C.A., Brauer B.E., Newman J.E., Tonelli A.R., Dweik R.A. (2020). Platelet glycolytic metabolism correlates with hemodynamic severity in pulmonary arterial hypertension. Am. J. Physiol. Lung Cell. Mol. Physiol..

[39] Nguyen Q.L., Corey C., White P., Watson A., Gladwin M.T., Simon M.A., Shiva S. (2017). Platelets from pulmonary hypertension patients show increased mitochondrial reserve capacity. JCI Insight.

[40] Culley M.K., Chan S.Y. (2018). Mitochondrial metabolism in pulmonary hypertension: Beyond mountains there are mountains. J. Clin. Investig..

[41] Yuan X., Zhang Y., Liu Y., Guo X., Jia S., Xiong X., Sun X., Jin Z. (2025). Multidimensional study on mitochondrial dysfunction in pulmonary hypertension. Front. Med..

[42] Liu X., Zhang L., Zhang W. (2022). Metabolic reprogramming: A novel metabolic model for pulmonary hypertension. Front. Cardiovasc. Med..

[43] Xu W., Janocha A.J., Xu W., Janocha A.J., Erzurum S.C. (2021). Metabolism in Pulmonary Hypertension. Annu. Rev. Physiol..

[44] Kelly B., O’Neill L.A. (2015). Metabolic reprogramming in macrophages and dendritic cells in innate immunity. Cell Res..

[45] Li M., Riddle S., Zhang H., D’Alessandro A., Flockton A., Serkova N.J., Hansen K.C., Moldovan R., McKeon B.A., Frid M. (2016). Metabolic Reprogramming Regulates the Proliferative and Inflammatory Phenotype of Adventitial Fibroblasts in Pulmonary Hypertension Through the Transcriptional Corepressor C-Terminal Binding Protein-1. Circulation.

[46] Chen Y., Lv Y., Yang M., Qian Z., Jiang X., Jiang Z., Kong C., Gu Y., Deng Y., Chen S.L. (2025). The P4HA1/HIF1α feedback loop modulates endothelial dysfunction in pulmonary hypertension. Respir. Res..

[47] Hua T., Zhang G., Yao Y., Jia H., Liu W. (2024). Research progress of megakaryocytes and platelets in lung injury. Ann. Med..

[48] Zhang W., Liu B., Wang Y., Zhang H., He L., Wang P., Dong M. (2022). Mitochondrial dysfunction in pulmonary arterial hypertension. Front. Physiol..

[49] Sekar D. (2021). Extracellular vesicles are involved in oxidative stress and mitochondrial homeostasis in pulmonary arterial hypertension. Hypertens. Res..

[50] Zhang J., Hu X., Wang T., Xiao R., Zhu L., Ruiz M., Dupuis J., Hu Q. (2023). Extracellular vesicles in venous thromboembolism and pulmonary hypertension. J. Nanobiotechnol..

[51] Kazimierczyk R., Kamiński K. (2018). The role of platelets in the development and progression of pulmonary arterial hypertension. Adv. Med. Sci..

[52] Sennett C., Pula G. (2025). Trapped in the NETs: Multiple Roles of Platelets in the Vascular Complications Associated with Neutrophil Extracellular Traps. Cells.

[53] Conti M., Minniti M., Tiné M., De Francesco M., Gaeta R., Nieri D., Semenzato U., Biondini D., Camera M., Cosio M.G. (2023). Extracellular Vesicles in Pulmonary Hypertension: A Dangerous Liaison?. Biology.

[54] Ohayon L., Zhang X., Dutta P. (2021). The role of extracellular vesicles in regulating local and systemic inflammation in cardiovascular disease. Pharmacol. Res..

[55] Kosanovic D., Deo U., Kosanovic D., Deo U., Gall H., Selvakumar B., Herold S., Weiss A., Petrovic A., Sydykov A. (2019). Enhanced circulating levels of CD3 cells-derived extracellular vesicles in different forms of pulmonary hypertension. Pulm. Circ..

[56] Ding Z., Qi F., Liu L., Wang Z., Zhang N., Lyu X., Sun W., Du J., Song H., Hou H. (2024). Circulating extracellular vesicles as novel biomarkers for pulmonary arterial hypertension in patients with systemic lupus erythematosus. Front. Immunol..

[57] Hasse S., Julien A.S., Duchez A.C., Zhao C., Boilard E., Fortin P.R., Bourgoin S.G. (2022). Red blood cell-derived phosphatidylserine positive extracellular vesicles are associated with past thrombotic events in patients with systemic erythematous lupus. Lupus Sci. Med..

[58] Mindubayeva F., Niyazova Y., Nigmatullina R., Kabiyeva S., Salikhova Y. (2020). The system of serotonin and its metabolism in platelets in children with congenital heart defect of an early age. Georgian Med. News.

[59] Mindubayeva F., Ospanova M., Akhmaltdinova L., Tukbekova B. (2024). Platelet-serotonin dynamics: Elucidating their role in pulmonary arterial hypertension. J. Clin. Med. Kaz..

[60] Ten Freyhaus H., Berghausen E.M., Janssen W., Leuchs M., Zierden M., Murmann K., Klinke A., Vantler M., Caglayan E., Kramer T. (2015). Genetic Ablation of PDGF-Dependent Signaling Pathways Abolishes Vascular Remodeling and Experimental Pulmonary Hypertension. Arterioscler. Thromb. Vasc. Biol..

[61] Ntokou A., Dave J.M., Kauffman A.C., Sauler M., Ryu C., Hwa J., Herzog E.L., Singh I., Saltzman W.M., Greif D.M. (2021). Macrophage-Derived PDGF-B Induces Muscularization in Murine and Human Pulmonary Hypertension. JCI Insight.

[62] Solinc J., Ribot J., Soubrier F., Pavoine C., Dierick F., Nadaud S. (2022). The Platelet-Derived Growth Factor Pathway in Pulmonary Arterial Hypertension: Still an Interesting Target?. Life.

[63] Good R.B., Gilbane A.J., Trinder S.L., Denton C.P., Coghlan G., Abraham D.J., Holmes A.M. (2015). Endothelial to Mesenchymal Transition Contributes to Endothelial Dysfunction in Pulmonary Arterial Hypertension. Am. J. Pathol..

[64] Cober N.D., VandenBroek M.M., Ormiston M.L., Stewart D.J. (2022). Evolving Concepts in Endothelial Pathobiology of Pulmonary Arterial Hypertension. Hypertension.

[65] Shen Y.H., Ding D., Lian T.Y., Qiu B.C., Yan Y., Wang P.W., Zhang W.H., Jing Z.C. (2024). Panorama of artery endothelial cell dysfunction in pulmonary arterial hypertension. J. Mol. Cell. Cardiol..

[66] Dai J., Chen H., Fang J., Wu S., Jia Z. (2025). Vascular Remodeling: The Multicellular Mechanisms of Pulmonary Hypertension. Int. J. Mol. Sci..

[67] Zuo Y., Li B., Gao M., Xiong R., He R., Li N., Geng Q. (2024). Novel insights and new therapeutic potentials for macrophages in pulmonary hypertension. Respir. Res..

[68] Lareo A., Nuche J., Cristo Ropero M.J., Arribas Ynsaurriaga F., Oliver E., Escribano-Subías P. (2021). Recent advances in the pharmacotherapy of pulmonary hypertension: Practical considerations. Kardiol. Pol..

[69] Martin de Miguel I., Cruz-Utrilla A., Oliver E., Escribano-Subias P. (2023). Molecular Mechanisms Involved in the Medical Treatment of Pulmonary Arterial Hypertension. Int. J. Mol. Sci..

[70] Erickson-Keith B. (2026). Case Report: Rethinking pulmonary arterial hypertension: Immune and metabolic adaptations in a 34-year case of insidious progression. Front. Cardiovasc. Med..

[71] Fernandes C.J.C.D.S., Nascimento I.A.O., Oliveira T., Jardim C.V.P., Hoette S., de Souza R. (2025). Prognostic Role of Platelets in Pulmonary Arterial Hypertension. Pulm. Circ..

[72] Bellan M., Giubertoni A., Piccinino C., Dimagli A., Grimoldi F., Sguazzotti M., Burlone M.E., Smirne C., Sola D., Marino P. (2019). Red Cell Distribution Width and Platelet Count as Biomarkers of Pulmonary Arterial Hypertension in Patients with Connective Tissue Disorders. Dis. Markers.

[73] Li Y., Liu X., Hong Q., Xu R. (2024). Platelet indices and the risk of pulmonary arterial hypertension: A two-sample and multivariable Mendelian randomization study. Front. Cardiovasc. Med..

[74] Wang L., Shen L., Zhao Y.L., Pudasaini B., Zhao Q.H., Gong S.G., Zhang R., Yuan P., He J., Luo C.J. (2021). Survival in severe pulmonary hypertension due to chronic lung disease: Influence of in-hospital platelet distribution width. Pulm. Circ..

[75] Zheng Y.G., Yang T., Xiong C.M., He J.G., Liu Z.H., Gu Q., Zhao Z.H., Ni X.H. (2015). Platelet distribution width and mean platelet volume in idiopathic pulmonary arterial hypertension. Heart Lung Circ..

[76] Mohamed M.F., Ali A., Abbas A., Awad M.S., Gouda M., Sediq A.M. (2019). Mean platelet volume as a predictor of pulmonary hypertension in patients with stable COPD. Int. J. Chron. Obstruct. Pulmon. Dis..

[77] Le R.J., Larsen C.M., Fenstad E.R., McCully R.B., Frantz R.P., McGoon M.D., Kane G.C. (2019). Thrombocytopenia independently predicts death in idiopathic PAH. Heart Lung.

[78] Grześk G., Karasek D., Kusiak M. (2020). Thrombocytopenia During Prostacyclin Analogue Therapies of Pulmonary Arterial Hypertension-Possible Pathomechanisms and Implications. J. Cardiovasc. Pharmacol..

[79] Balko J., Havlin J., CasasMendez F., Zajacova A., Koblizek M., Svorcova M., Lischke R., Zamecnik J. (2022). Mapping of the lung megakaryocytes: A role in pathogenesis of idiopathic pulmonary arterial hypertension?. Pathol. Res. Pract..

[80] Christman B.W., McPherson C.D., Newman J.H., King G.A., Bernard G.R., Groves B.M., Loyd J.E. (1992). An imbalance between the excretion of thromboxane and prostacyclin metabolites in pulmonary hypertension. N. Engl. J. Med..

[81] Gąsecka A., Banaszkiewicz M., Nieuwland R., van der Pol E., Hajji N., Mutwil H., Rogula S., Rutkowska W., Pluta K., Eyileten C. (2021). Prostacyclin Analogues Inhibit Platelet Reactivity, Extracellular Vesicle Release and Thrombus Formation in Patients with Pulmonary Arterial Hypertension. J. Clin. Med..

[82] Clapp L.H., Abu-Hanna J.H.J., Patel J.A. (2020). Diverse pharmacology of prostacyclin mimetics: Implications for pulmonary hypertension. Molecular Mechanism of Congenital Heart Disease and Pulmonary Hypertension.

[83] Siniarski A., Gąsecka A., Starczyński M., Banaszkiewicz M., Darocha S., Torbicki A., Kurzyna M., Filipiak K.J., Nessler J., Gajos G. (2022). Prostacyclin analogues decrease platelet aggregation but have no effect on thrombin generation, fibrin clot structure, and fibrinolysis in pulmonary arterial hypertension: PAPAYA coagulation. Platelets.

[84] Degjoni A., Campolo F., Stefanini L., Venneri M.A. (2022). The NO/cGMP/PKG pathway in platelets: The therapeutic potential of PDE5 inhibitors in platelet disorders. J. Thromb. Haemost..

[85] Barnes H., Brown Z., Burns A., Williams T. (2019). Phosphodiesterase 5 inhibitors for pulmonary hypertension. Cochrane Database Syst. Rev..

[86] Andersson K.E. (2018). PDE5 inhibitors—Pharmacology and clinical applications 20 years after sildenafil discovery. Br. J. Pharmacol..

[87] Humbert M., Ghofrani H.-A. (2016). The molecular targets of approved treatments for pulmonary arterial hypertension. Thorax.

[88] Grześk G., Nowaczyk A. (2021). Current Modulation of Guanylate Cyclase Pathway Activity-Mechanism and Clinical Implications. Molecules.

[89] Robinson J.C., Pugliese S.C., Robinson J.C., Pugliese S.C., Fox D.L., Badesch D.B. (2016). Anticoagulation in Pulmonary Arterial Hypertension. Curr. Hypertens. Rep..

[90] Khan M.S., Usman M.S., Siddiqi T.J., Khan S.U., Murad M.H., Mookadam F., Figueredo V.M., Krasuski R.A., Benza R.L., Rich J.D. (2018). Is Anticoagulation Beneficial in Pulmonary Arterial Hypertension?. Circ. Cardiovasc. Qual. Outcomes.

[91] Lu M., Blaine K.P., Cullinane A., Hall C., Dulau-Florea A., Sun J., Chenwi H.F., Graninger G.M., Harper B., Thompson K. (2021). Pulmonary arterial hypertension patients display normal kinetics of clot formation using thrombelastography. Pulm. Circ..

[92] Saleh K.M., Mallat J., Mohammed S., Bodi G., Alazazzi H., Salim S., Elhennawi M., Iqbal T., Sabbour H. (2025). Comparative efficacy and safety of prostacyclin therapies for pulmonary arterial hypertension: A systematic review and network meta-analysis. Front. Med..

